# Secular Trends in the Burden of Multiple Myeloma From 1990 to 2019 and Its Projection Until 2044 in China

**DOI:** 10.3389/fpubh.2022.938770

**Published:** 2022-07-08

**Authors:** Yumei Zhao, Dongdong Niu, Enlin Ye, Jiasheng Huang, Jia Wang, Xuefei Hou, Jiayuan Wu

**Affiliations:** ^1^Clinical Research Service Center, Affiliated Hospital of Guangdong Medical University, Zhanjiang, China; ^2^Guangdong Engineering Research Center of Collaborative Innovation Technology of Clinical Medical Big Data Cloud Service in Medical Consortium of West Guangdong Province, Affiliated Hospital of Guangdong Medical University, Zhanjiang, China

**Keywords:** multiple myeloma, disease burden, secular trend, projection, age-period-cohort model

## Abstract

**Objective:**

Multiple myeloma (MM) imposes a heavy burden in China. Understanding the secular trend of MM burden and projecting its future trend could facilitate appropriate public health planning and improve the management of MM.

**Methods:**

Sex-specific incidence and mortality rates of MM in China from 1990 to 2019 were collected from the Global Burden of Disease 2019 study. The secular trend of MM burden was analyzed by joinpoint regression. Age–period–cohort model was used to analyze the effects of age, period, and birth cohort on MM burden and project future trends up to 2044.

**Results:**

From 1990 to 2019, the age-standardized incidence and mortality rates of MM continued to increase in males. For females, the age-standardized rates were stable in MM incidence and decreased in MM mortality. Males had a higher disease burden of MM than females. Age effects were the most significant risk factor for MM incidence and mortality. Moreover, the risk of MM incidence and mortality increased with increasing time period but decreased with birth cohort in males and females. The age-standardized incidence and mortality rates of MM in China is predicted to be continuously increasing over the next 25 years.

**Conclusion:**

The burden of MM in China is expected to continue to increase in the future, with significant sex difference. A comprehensive understanding of the risk characteristics and disease pattern of MM could help develop timely intervention measures to effectively reduce its burden.

## Introduction

Multiple myeloma (MM) is a malignant proliferative disease of plasma cells. It is characterized by abnormal proliferation of clonal plasma cells in bone marrow. In most cases, monoclonal immunoglobulin or its fragment (M-protein) is secreted, resulting in damage to related organs or tissues. The common clinical manifestations of MM are bone pain, anemia, renal function damage, hypercalcemia and infection. According to the GLOBOCAN 2020 statistics, 176,404 new cases of MM were reported worldwide in 2020, ranking 21nd among all cancers; MM also led to 117,077 deaths, ranking 17th among leading causes of cancer death (1). In 2020, the estimated numbers of new MM cases and deaths in China were 32,000 and 27,600, ranking the 21nd and 18th leading causes of cancer diagnosis and mortality in China, respectively (1). This finding indicated that China is facing a serious disease burden of MM (1, 2). A comprehensive understanding of MM epidemiology is the basic requirement to promote effective intervention measures and the rational allocation of healthcare resources (2, 3).

The diagnostic criteria and techniques of MM are constantly being updated and improved. Meanwhile, the treatment of MM continues to evolve rapidly with the arrival of multiple new drugs and emerging data from randomized trials to guide therapy (4). The 5-year survival rate of young patients with MM has increased from 74.1% in 2010 to 78.5% in 2016 (5). Early diagnosis and timely treatment could considerably improve the survival of patients with MM. Despite great advances in diagnosis and treatment of MM over the past few decades, knowledge of MM etiology and its potential mechanism remain lacking.

Studies have suggested that ionizing radiation, overweight and obesity, lack of nutrients and unhealthy lifestyle are risk factors for MM, and these factors may vary with chronological age, time period and birth cohort (6–8). However, few studies in China specifically analyzed the trends of MM incidence and mortality rates by age, period and birth cohort (9). Age effects exhibit different risks associated with different age brackets. Period effects refer to the effects of a complex combination of historical events and environmental factors. Birth cohort effects represent the influence of physical and social exposure in early life that may accumulate over time. Therefore, by simultaneously reflecting the net age, period (year of survey) and cohort (year of birth) effects of long-term trends in disease, the age–period–cohort model has an advantage in assessing the disease burden and provide information on the etiology of MM. This study aimed to provide a comprehensive assessment on the secular trend of MM incidence and mortality in China from 1990 to 2019 under the age–period–cohort framework and project their trends in the next 25 years.

## Data and Methods

### Data Sources

Data on the sex-specific disease burden of MM in China from 1990 to 2019 were obtained from the official website of the Global Burden of Disease (GBD) 2019 Study (http://ghdx.healthdata.org/gbdresults-tool). The GBD 2019 database provides a comprehensive assessment of the incidence rate, mortality, life lost years, and disability adjusted life years of 369 diseases and injuries and 87 risk factors in 204 countries and 21 regions from 1990 to 2019. Details on the data, statistical modeling, and metrics for GBD 2019 have been reported in previous studies (10, 11). The sources of disease death data in China are mainly the national disease surveillance system, maternal and child surveillance system, and national cause of death reporting system. “China” was chosen from the database as the location, “multiple myeloma” for the cause, and “deaths” and “incidence” for measures. In this study, the age-standardized rates (ASRs), the crude rates and the absolute number of MM incidence and mortality were also extracted by sex and age. The ASRs and 95% uncertainty intervals (UIs) were calculated on the basis of GBD 2019 global age-standard population (12).

### Joinpoint Analysis

Joinpoint regression model is used to describe the continuous change in the slopes of overall trend through displacement analysis, and it divides a secular trend line into several statistically significant trend section by model fitting. In this model, the average annual percent change (AAPC) and annual percent change (APC) were calculated. The AAPC or APC > 0 denotes an increasing trend, whereas the AAPC or APC < 0 represents a decreasing trend, which only makes sense if their 95% confidence intervals (CIs) upper limit and lower limit have the same sign. Otherwise, the trend was regarded as stable over time. The Joinpoint Regression Program 4.7.0.0 software was used for joinpoint regression analysis.

### Age–Period–Cohort Model Analysis

An age–period–cohort model was developed on the basis of Poisson distribution, which could simultaneously estimate the influence of age, period and birth cohort on the trends. The APC model could be expressed as follows:


$$\begin{array}{r} \log\left(Eij\right)=\log\left(Pij\right)+\mu +\alpha i+\beta j+\gamma k. \end{array}$$

The age–period–cohort model requires an equal time interval in age, period, and cohort. Otherwise, an information overlap in the adjacent queues could occur. GBD 2019 assumed no newly diagnosed cases prior to age 20 for MM. These settings are in line with the corresponding cause of death model for MM. The sex-specific rates were appropriately recorded into successive 5-year groups (20–24, 25–29… 95+ years), consecutive 5-year period from 1990 to 2019, and correspondingly consecutive 5-year birth cohort groups starting from 1895–1899 to 1995–1999.

As the cohort is obtained by subtracting the corresponding age from the period (cohort = period – age), a collinearity problem exists among the age, period and cohort. When estimating the age, period, and cohort effects independently, the parameters are not identifiable. Intrinsic estimator (IE) is introduced when using the parameter estimation of the APC model to solve this problem (13, 14). The age–period–cohort analysis with the IE method provided estimated coefficients for the age, period and cohort effects. These coefficients were transformed into the exponential value [exp(coef.) = e^coef.^], which denotes the relative risk (RR) of a particular age, period or birth cohort relative to the average level of all ages, periods or birth cohorts combined. For example, for the age effect in males, the RR of MM incidence in people aged 85–89 years was 3.29, which indicated that the risk in this age group was 3.29 times greater than the risk for all ages combined. The age–period–cohort analysis was performed using STATA 15.0 software.

### Bayesian Age-Period-Cohort (BAPC) Analysis

The ASRs of the MM incidence and death in China from 2020 to 2044 were also predicted. Bayesian age-period-cohort (BAPC) analysis was conducted based on the assumption that the effects of age, period, and cohort adjacent in time are similar, which applies the second-order random walk for smoothing priors of age, period, and cohort effects and to project posterior incidence and mortality rates. In order to avoid any mixing and convergence issues caused by Markov chain Monte Carlo sampling techniques traditionally used in the Bayesian approach. The integrated nested Laplace approximations (INLA) are introduced when using the Bayesian age-period-cohort model to approximate the marginal posterior distributions. Which shows better coverage and precision than other prediction methods (15, 16). The BAPC analysis was performed using R-package BAPC. The population predictions for China were taken from the 2019 revision of the United Nations (UN) World Population Prospects and were used to estimate China's population in 2020 and beyond.

## Results

### Descriptive Analysis

The sex-specific incidence and death rates of MM by age groups in China in 2019 are listed in Table 1. In 2019, the age-standardized incidence rates (ASIRs) of MM in China were 1.16 (95% UI: 0.77–1.54) and 0.72 (95% UI: 0.49–0.94) per 100,000 population in males and females, respectively. The age-standardized mortality rates (ASMRs) of MM in China were 0.83 (95% UI: 0.54–1.08) and 0.55 (95% UI: 0.39–0.71) per 100,000 population in males and females, respectively. The males had higher ASRs of incidence and mortality than the females in all patients and each age group. For males, the highest ASIR and ASMR were observed in the group aged 85–89 years, followed by the groups aged 90–94 years. For females, the highest ASIR and ASMR were observed in the group aged 75–79 years, followed by the groups aged 70–74 years.

**Table 1:** The sex-age-specific rates of MM in China in 2019 and their percentage changes from 1990 to 2019.

**Categories**	**Males**		**Females**	
	**Rates in 2019, 95% UI** **(per 100 000 population)**	**AAPC, 95% CI** **(%, 1990–2019)**	**Rates in 2019, 95% UI** **(per 100 000 population)**	**AAPC, 95% CI** **(%, 1990–2019)**
**Incidence**				
ASR	1.16 (0.77–1.54)	1.85 (1.76–1.95) *	0.72 (0.49–0.94)	0.10 (0.03–0.18) *
20–24 years	0.28 (0.10–0.42)	4.18 (3.53–4.83) *	0.14 (0.05–0.22)	2.66 (1.46–3.88) *
25–29 years	0.26 (0.11–0.38)	4.42 (4.00–4.84) *	0.11 (0.04–0.17)	1.54 (0.20–2.98) *
30–34 years	0.24 (0.13–0.32)	2.64 (2.39–2.89) *	0.13 (0.06–0.20)	1.08 (−0.13–2.30)
35–39 years	0.39 (0.22–0.52)	1.65 (1.37–1.93) *	0.21 (0.10–0.31)	0.55 (−0.01–1.12)
40–44 years	0.57 (0.37–0.79)	1.31 (1.16–1.47) *	0.35 (0.19–0.48)	0.29 (−0.02–0.60)
45–49 years	0.92 (0.61–1.28)	1.73 (1.52–1.94) *	0.55 (0.33–0.75)	−0.21 (−0.62–0.20)
50–54 years	1.70 (1.13–2.38)	1.24 (1.15–1.33) *	1.10 (0.69–1.47)	−0.17 (−0.26–−0.07) *
55–59 years	2.77 (1.84–3.93)	1.12 (1.04–1.21) *	1.85 (1.24–2.52)	−0.03 (−0.36–0.30)
60–64 years	4.09 (2.69–5.65)	1.67 (1.50–1.84) *	2.68 (1.77–3.58)	0.00 (−0.19–0.19)
65–69 years	5.50 (3.58–7.51)	1.83 (1.68–1.98) *	3.83 (2.66–5.00)	0.05 (−0.10–0.20)
70–74 years	7.30 (4.74–9.93)	2.08 (1.96–2.21) *	5.10 (3.57–6.85)	0.24 (0.16–0.31) *
75–79 years	7.48 (5.02–9.84)	2.12 (2.00–2.25) *	5.26 (3.78–7.00)	0.15 (0.05–0.25) *
80–84 years	7.90 (5.39–10.38)	1.99 (1.87–2.10) *	4.20 (2.98–5.64)	0.24 (0.13–0.34) *
85–89 years	11.57 (7.50–14.87)	2.42 (2.13–2.72) *	3.58 (2.55–4.88)	−0.24 (-0.43–−0.04) *
90–94 years	9.32 (6.01–12.11)	2.56 (2.22–2.91) *	3.84 (2.76–5.30)	0.25 (0.05–0.45) *
95+ years	5.61 (3.66–7.38)	1.95 (1.61–2.28) *	2.73 (1.84–3.77)	0.47 (0.25–0.68) *
**Mortality**				
ASR	0.83 (0.54–1.08)	0.93 (0.85–1.00) *	0.55 (0.39–0.71)	−0.64 (−0.71–−0.56)
20–24 years	0.12 (0.05–0.19)	3.00 (2.31–3.70) *	0.07 (0.02–0.10)	1.62 (0.39–2.86) *
25–29 years	0.12 (0.05–0.18)	3.12 (2.67–3.58) *	0.06 (0.02–0.09)	0.52 (−0.85–1.90)
30–34 years	0.13 (0.07–0.17)	1.50 (1.22–1.79) *	0.08 (0.03–0.12)	0.1 (−1.14–1.35)
35–39 years	0.22 (0.12–0.29)	0.53 (0.23–0.84) *	0.13 (0.06–0.18)	−0.40 (−0.99–0.19)
40–44 years	0.35 (0.22–0.47)	0.21 (0.03–0.39) *	0.22 (0.13–0.31)	−0.65 (−0.97–−0.32) *
45–49 years	0.53 (0.34–0.74)	0.63 (0.46–0.81) *	0.34 (0.20–0.46)	−1.15 (−1.57–−0.72) *
50–54 years	0.98 (0.62–1.38)	0.13 (0.06–0.20) *	0.68 (0.45–0.91)	−1.11 (−1.21–−1.00) *
55–59 years	1.66 (1.09–2.34)	0.05 (-0.04–0.14)	1.18 (0.80–1.56)	−0.93 (−1.23–−0.62) *
60–64 years	2.67 (1.67–3.73)	0.70 (0.58–0.83) *	1.87 (1.28–2.49)	−0.78 (−0.95 to −0.61) *
65–69 years	3.82 (2.43–5.17)	0.90 (0.79–1.02) *	2.90 (2.08–3.79)	−0.62 (−0.77 to −0.48) *
70–74 years	5.39 (3.43–7.17)	1.17 (1.08–1.26) *	4.06 (2.92–5.39)	−0.45 (−0.53 to −0.38) *
75–79 years	6.22 (4.09–8.14)	1.26 (1.17–1.36) *	4.81 (3.48–6.30)	−0.41 (−0.50 to −0.32) *
80–84 years	7.03 (4.73–9.10)	1.12 (1.02–1.22) *	4.02 (2.92–5.38)	−0.39 (−0.48 to −0.30) *
85–89 years	11.09 (7.15–13.91)	1.56 (1.26–1.86) *	3.63 (2.59–4.94)	−0.87 (−1.08 to −0.66) *
90–94 years	9.99 (6.49–12.68)	1.65 (1.30–2.00) *	4.36 (3.08–5.80)	−0.45 (−0.67 to −0.22) *
95+ years	7.06 (4.66–9.39)	0.98 (0.61–1.36) *	3.81 (2.72–5.12)	−0.14 (−0.38 to 0.10)

* *Indicates the AAPC was significantly different from zero at the α = 0.05 level. UI, uncertainty interval; CI, confidence interval; ASR, age–standardized rates; AAPC, average annual percent change*.

### Jointpoint Regression Analysis

The AAPCs of the age-sex-specific rates of MM incidence and mortality from 1990 to 2019 are shown in Table 1. For males, the ASIRs and ASMRs of MM in China from 1990 to 2019 annually rose by 1.85% (95% CI: 1.76–1.95) and 0.93% (95% CI: 0.85–1.00), respectively. For females, the ASIRs showed an increasing trend, with AAPC of 0.10% (95% CI: 0.03–0.18), whereas the ASMRs presented a decreasing trend, with AAPC of −0.64% (95% CI: from −0.71 to −0.56) during the observation period.

**Figure 1:**
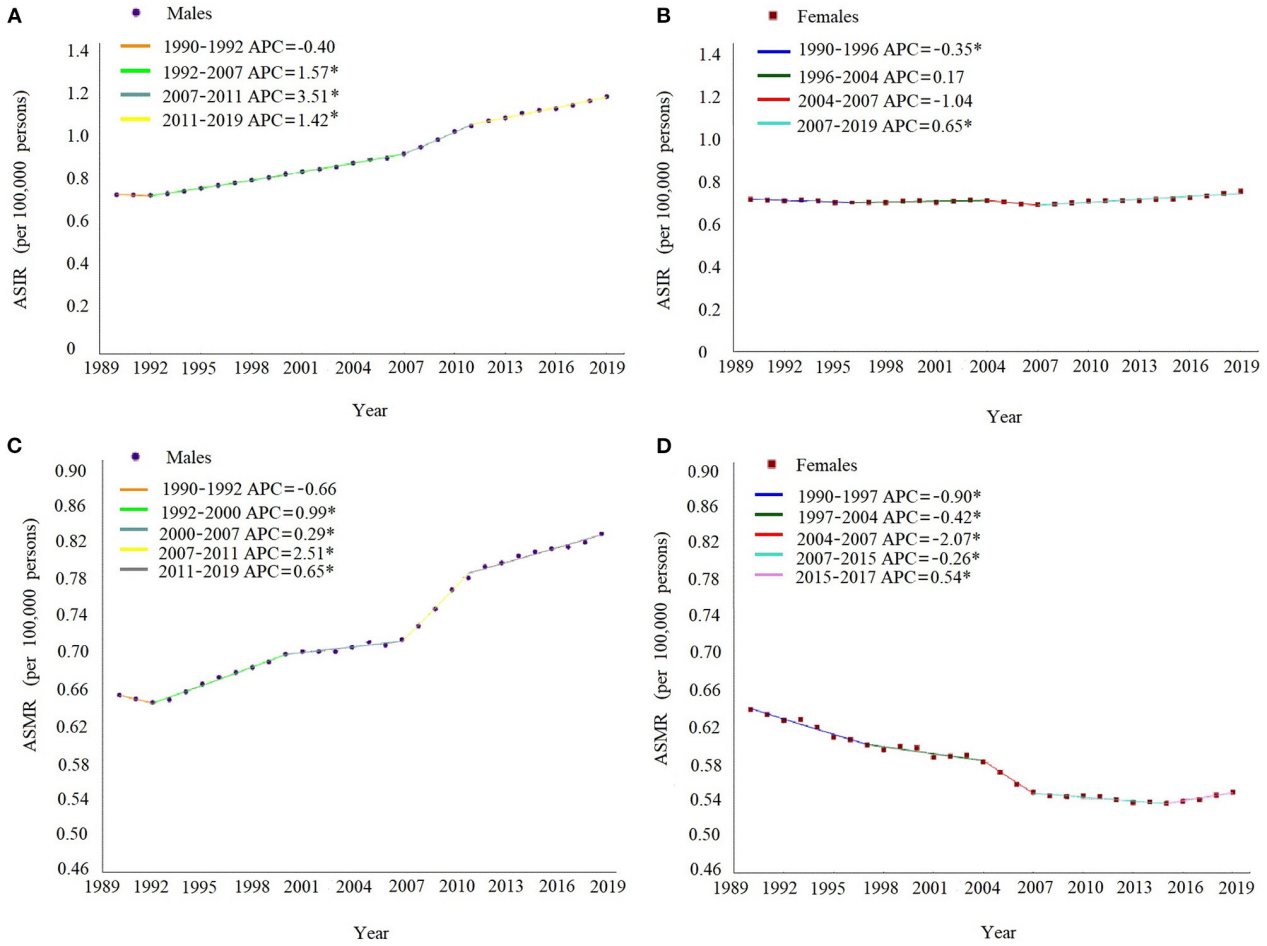
Joinpoint regression analysis in sex-specific age-standardized incidence and death rates of multiple myeloma in China from 1990 to 2019. The ASIR in males **(A)**, ASIR in females **(B)**, ASMR in males **(C)**, and ASMR in females **(D)**. Notes: the annual percent change is statistically significantly different from zero at the a = 0.05 level. ASIR, age-standardized incidence rates. ASMR, age-standardized death rates.

As shown in Figure 1, during 1990–2019, the temporal trend of sex-specific ASIRs of MM contained three turning points and were divided into four sections, while the secular trend of ASMRs contained four turning points and were divided into five sections in both sexes. For males, the ASIRs and ASMRs continuously increased after 1992, with the most significant increase during the period of 2007–2011 (APC = 3.51, 95% CI: 3.04–3.98 for ASIR; APC = 2.51, 95% CI: 2.06–2.96 for ASMR). For females, the ASIRs continuously increased after 2007 (APC = 0.65, 95% CI: 0.53–0.76), whereas the ASMRs showed a significant decrease during the period of 1990–2015, with the most significant decrease during the period of 2004–2007 (APC = −2.07, 95% CI: from −3.35 to −0.78), as shown in Supplementary Table 1.

### Age–Period–Cohort Analysis

The estimated RRs of MM incidence and mortality due to age, period and cohort effects are shown in Table 2. When the period and cohort effects were controlled, the RRs of age effects on MM incidence sharply increased from 0.20 (95% CI: 0.05–0.35) in the group aged 20–24 years to 3.29 (95% CI: 2.21–4.40) in the group aged 85–89 years for males, and from 0.21 (95% CI: 0.05–0.40) in the group aged 20–24 years to 3.55 (95% CI: 2.15–4.98) in the group aged 70–74 years for females. Then, the RRs continuously decreased with increasing age in males and females. Time period had a significant effect on MM incidence in males, with the RRs increased from 0.62 (95% CI: 0.41–0.93) in 1994 to 1.62 (95% CI: 1.14–2.20) in 2019. For cohort effects, the RRs due to cohort effects continuously decreased from 2.52 (95% CI 1.72–3.33) in the cohort 1895–1899 to 0.54 (95% CI 0.06–1.03) in the cohort 1995–1999 for males, and from 2.31 (95% CI 1.53–3.18) in the cohort 1895–1899 to 0.46 (95% CI 0.08–0.87) in the cohort 1995–1999 for females.

**Table 2:** Sex – specific relative risks of MM in China due to age, period, and cohort effects.

**Categories**	**Males**				**Females**			
	**Incidence**		**Mortality**		**Incidence**		**Mortality**	
	**RR (95% CI)**	***P*-value**	**RR (95% CI)**	***P*-value**	**RR (95% CI)**	***P*-value**	**RR (95% CI)**	***P*-value**
**Age effects**								
20–24	0.20 (0.06–0.35)	<0.001	0.25 (0.10–0.40)	<0.001	0.21 (0.05–0.40)	<0.001	0.26 (0.11–0.44)	<0.001
25–29	0.19 (0.07–0.34)	<0.001	0.25 (0.12–0.40)	<0.001	0.17 (0.07–0.30)	<0.001	0.34 (0.18–0.51)	<0.001
30–34	0.19 (0.08–0.33)	<0.001	0.36 (0.20–0.54)	0.014	0.20 (0.09–0.33)	<0.001	0.38 (0.21–0.53)	<0.001
35–39	0.30 (0.10–0.50)	<0.001	0.56 (0.28–0.92)	0.046	0.28 (0.11–0.48)	<0.001	0.49 (0.25–0.75)	<0.001
40–44	0.44 (0.12–0.77)	<0.001	0.61 (0.31–1.08)	0.087	0.42 (0.14–0.75)	<0.001	0.66 (0.32–1.03)	0.069
45–49	0.67 (0.23–1.15)	0.458	0.78 (0.35–1.22)	0.127	0.67 (0.19–1.22)	0.332	0.79 (0.53–1.15)	0.488
50–54	1.07 (0.44–1.75)	0.876	0.92 (0.40–1.39)	0.287	1.15 (0.41–1.97)	0.793	0.98 (0.69–1.30)	0.674
55–59	1.58 (0.75–2.34)	0.230	1.39 (0.55–2.27)	0.484	1.71 (0.73–2.92)	0.416	1.47 (0.83–2.07)	0.262
60–64	2.03 (1.08–3.11)	0.028	1.88 (0.88–2.91)	0.105	2.27 (1.11–3.37)	0.025	2.07 (1.19–3.21)	0.005
65–69	2.40 (1.41–3.48)	0.001	2.32 (1.24–3.44)	0.008	2.93 (1.61–4.33)	<0.001	2.84 (1.43–4.33)	0.003
70–74	2.74 (1.76–3.78)	<0.001	2.79 (1.67–3.96)	<0.001	3.55 (2.15–4.98)	<0.001	3.27 (2.03–4.40)	<0.001
75–79	2.69 (1.80–3.62)	<0.001	3.00 (1.91–4.11)	<0.001	3.42 (2.15–4.75)	<0.001	3.46 (2.31–3.54)	<0.001
80–84	2.56 (1.73–3.40)	<0.001	3.04 (1.95–4.14)	<0.001	2.46 (1.49–3.56)	<0.001	2.91 (1.67–3.26)	<0.001
85–89	3.29 (2.21–4.40)	<0.001	4.16 (2.60–5.67)	<0.001	1.99 (1.13–2.88)	0.017	2.49 (1.32–3.68)	0.005
90–94	2.48 (1.54–3.50)	<0.001	3.49 (1.97–5.99)	<0.001	1.87 (0.99–2.72)	0.054	2.61 (1.25–4.03)	0.010
95–99	1.27 (0.68–2.06)	0.453	2.08 (1.01–3.00)	0.047	1.17 (0.52–2.10)	0.704	1.98 (0.80–3.17)	0.138
**Period effects**								
1994	0.62 (0.41–0.93)	0.021	0.77 (0.42–1.08)	0.097	0.79 (0.50–1.11)	0.307	0.83 (0.49–1.22)	0.505
1999	0.76 (0.56–1.05)	0.094	0.92 (0.58–1.16)	0.256	0.87 (0.60–1.18)	0.440	0.91 (0.61–1.25)	0.635
2004	0.86 (0.64–1.13)	0.291	0.98 (0.69–1.28)	0.358	0.94 (0.69–1.19)	0.711	0.95 (0.69–1.32)	0.780
2009	1.09 (0.84–1.42)	0.501	1.10 (0.81–1.39)	0.674	1.02 (0.74–1.41)	0.900	1.00 (0.71–1.30)	0.989
2014	1.39 (1.04–1.85)	0.028	1.31 (0.94–1.73)	0.113	1.15 (0.80–1.57)	0.444	1.11 (0.74–1.47)	0.622
2019	1.62 (1.14–2.20)	0.007	1.50 (0.97–2.04)	0.069	1.32 (0.86–1.84)	0.202	1.25 (0.89–1.59)	0.394
**Cohort effects**								
1895–1899	2.52 (1.72–3.33)	<0.001	2.73 (1.06–4.47)	0.040	2.31 (1.53–3.18)	<0.001	2.60 (0.73–4.53)	0.388
1900–1904	2.33 (1.61–3.06)	<0.001	2.55 (1.08–4.02)	0.033	2.28 (1.42–3.04)	<0.001	2.57 (0.89–4.35)	0.181
1905–1909	2.13 (1.41–2.88)	<0.001	2.38 (1.16–3.58)	0.018	2.15 (1.25–3.06)	<0.001	2.38 (1.00–3.77)	0.050
1910–1914	1.87 (1.29–2.52)	<0.001	2.06 (1.14–3.03)	0.017	1.93 (1.08–2.80)	0.017	2.12 (1.03–3.28)	0.041
1915–1919	1.67 (1.16–2.15)	0.008	1.83 (1.11–2.61)	0.017	1.72 (0.97–2.75)	0.062	1.88 (1.02–2.77)	0.043
1920–1924	1.64 (1.10–2.12)	0.014	1.78 (1.15–2.34)	0.009	1.56 (0.94–2.20)	0.086	1.70 (0.97–2.45)	0.062
1925–1929	1.50 (1.00–2.04)	0.050	1.61 (1.02–2.24)	0.043	1.44 (0.85–2.04)	0.171	1.55 (0.86–2.30)	0.143
1930–1934	1.37 (0.86–1.87)	0.181	1.46 (0.85–2.05)	0.174	1.33 (0.74–1.90)	0.339	1.43 (0.72–2.12)	0.307
1935–1939	1.24 (0.71–1.74)	0.451	1.30 (0.67–1.93)	0.437	1.22 (0.62–1.84)	0.568	1.30 (0.58–2.05)	0.525
1940–1944	1.10 (0.58–1.65)	0.567	1.15 (0.52–1.76)	0.727	1.08 (0.50–1.65)	0.642	1.15 (0.45–1.93)	0.776
1945–1949	1.02 (0.48–1.66)	0.659	1.04 (0.41–1.67)	0.930	1.02 (0.41–1.60)	0.770	1.05 (0.35–1.68)	0.825
1950–1954	0.88 (0.37–1.41)	0.480	0.89 (0.30–1.77)	0.835	0.93 (0.33–1.53)	0.691	0.95 (0.26–1.63)	0.936
1955–1959	0.77 (0.29–1.31)	0.414	0.76 (0.21–1.32)	0.577	0.82 (0.25–1.43)	0.649	0.82 (0.18–1.56)	0.794
1960–1964	0.68 (0.22–1.15)	0.316	0.66 (0.15–1.16)	0.376	0.74 (0.22–1.24)	0.474	0.72 (0.13–1.35)	0.452
1965–1969	0.62 (0.17–1.06)	0.164	0.58 (0.11–1.03)	0.128	0.67 (0.20–1.14)	0.219	0.64 (0.11–1.24)	0.256
1970–1974	0.53 (0.12–0.97)	0.048	0.49 (0.08–0.92)	0.036	0.59 (0.15–1.10)	0.166	0.55(0.10–1.11)	0.107
1975–1979	0.52 (0.09–0.91)	0.042	0.47 (0.08–0.88)	0.031	0.57 (0.12–1.05)	0.062	0.52 (0.07–0.99)	0.048
1980–1984	0.51 (0.08–0.94)	0.043	0.44 (0.06–0.82)	0.028	0.50 (0.10–0.90)	0.006	0.44 (0.06–0.88)	0.022
1985–1989	0.50 (0.07–0.93)	0.043	0.43 (0.06–0.80)	0.019	0.47 (0.09–0.84)	0.005	0.40 (0.05–0.78)	<0.001
1990–1994	0.51 (0.06–0.95)	0.048	0.43 (0.05–0.84)	0.016	0.47 (0.08–0.91)	0.010	0.39 (0.05–0.75)	<0.001
1995–1999	0.54 (0.06–1.03)	0.171	0.45 (0.05–0.86)	0.020	0.46 (0.08–0.87)	0.006	0.38 (0.04–0.72)	<0.001
Deviance	0.5		0.5		0.32		– 0.29	
AIC	3.35		3.19		3.04		2.93	
BIC	– 255.11		– 255.11		– 255.29		– 255.32	

*RR denotes the relative risk of MM incidence and deaths in a particular age, period, or birth cohort relative to the average level of all age, period, or birth cohort combined. RR, relative risk; CI, confidence interval; AIC, Akaike Information Criterion; BIC, Bayesian Information Criterion*.

**Figure 2:**
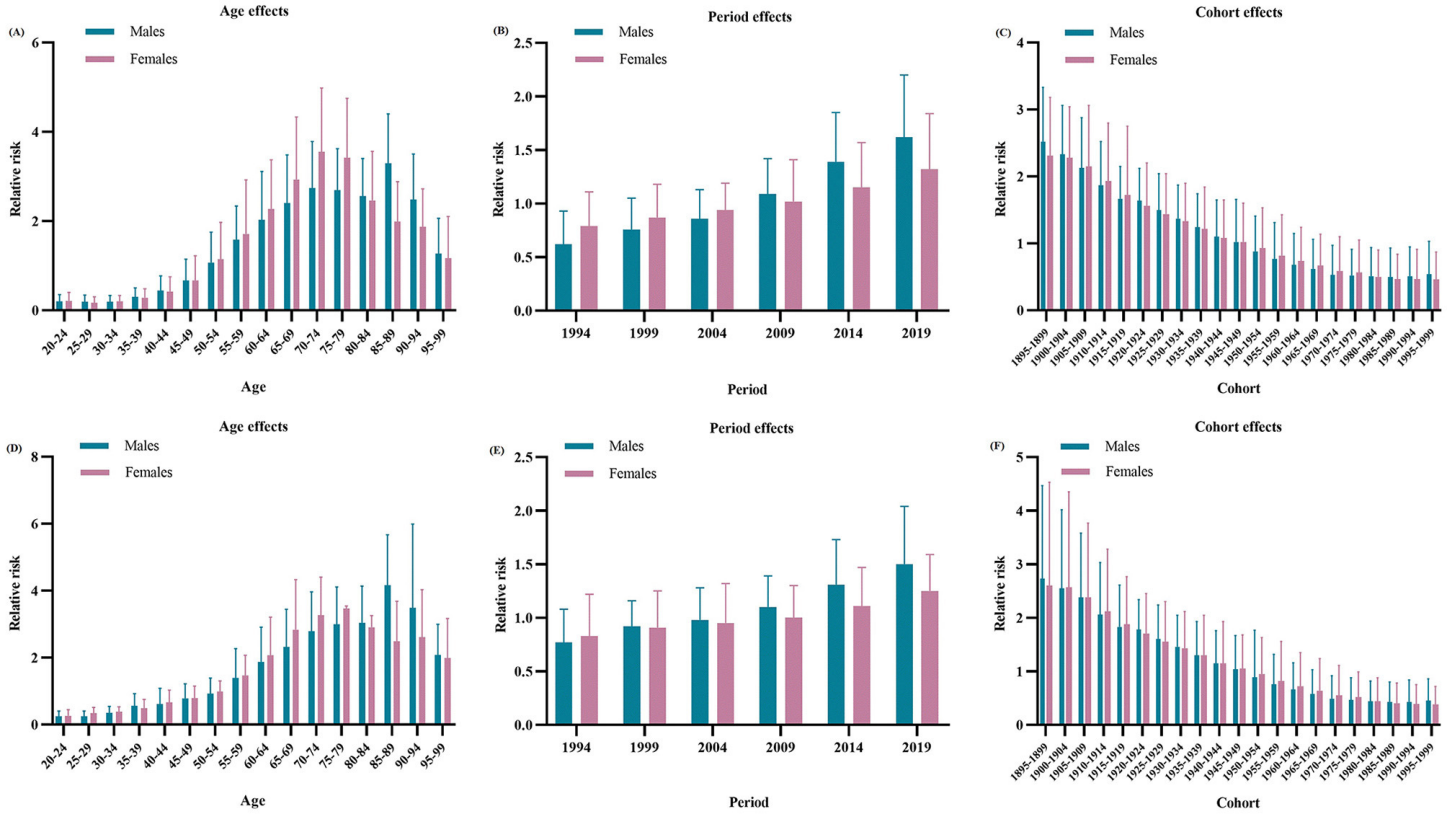
Relative risks of incidence due to age **(A)**, period **(B)**, and cohort **(C)** effects, and relative risks of deaths due to age **(D)**, period **(E)**, and cohort **(F)** effects of multiple myeloma in China from 1990 to 2019.

As shown in Figure 2, after adjustment by the period and cohort effects, the RRs due to age effects on MM mortality continuously increased from 0.25 (95% CI: 0.10–0.40) in the group aged 20–24 years to 4.16 (95% CI: 2.60–5.67) in the group aged 85–89 years for males, and from 0.26 (95% CI: 0.11–0.44) in the group aged 20–24 years to 3.46 (95% CI: 2.31–3.54) in the group aged 75–79 years for females. Then, the RRs slowly decreased with increasing age in males and females. However, time period had a non-significant effect on MM mortality in both sexes. For cohort effects, later birth cohorts experienced a relatively low risk of MM-related death compared with earlier cohorts. The RRs due to cohort effects decreased from 2.73 (95% CI: 1.06–4.47) in the cohort 1895–1899 to 0.45 (95% CI: 0.05–0.86) in the cohort 1995–1999 for males, and from 2.60 (95% CI: 0.73–4.53) in the cohort 1895–1899 to 0.38 (95% CI: 0.04–0.72) in the cohort 1995–1999 for females.

### Predictions of MM Burden in the Coming 25 Years in China

**Figure 3:**
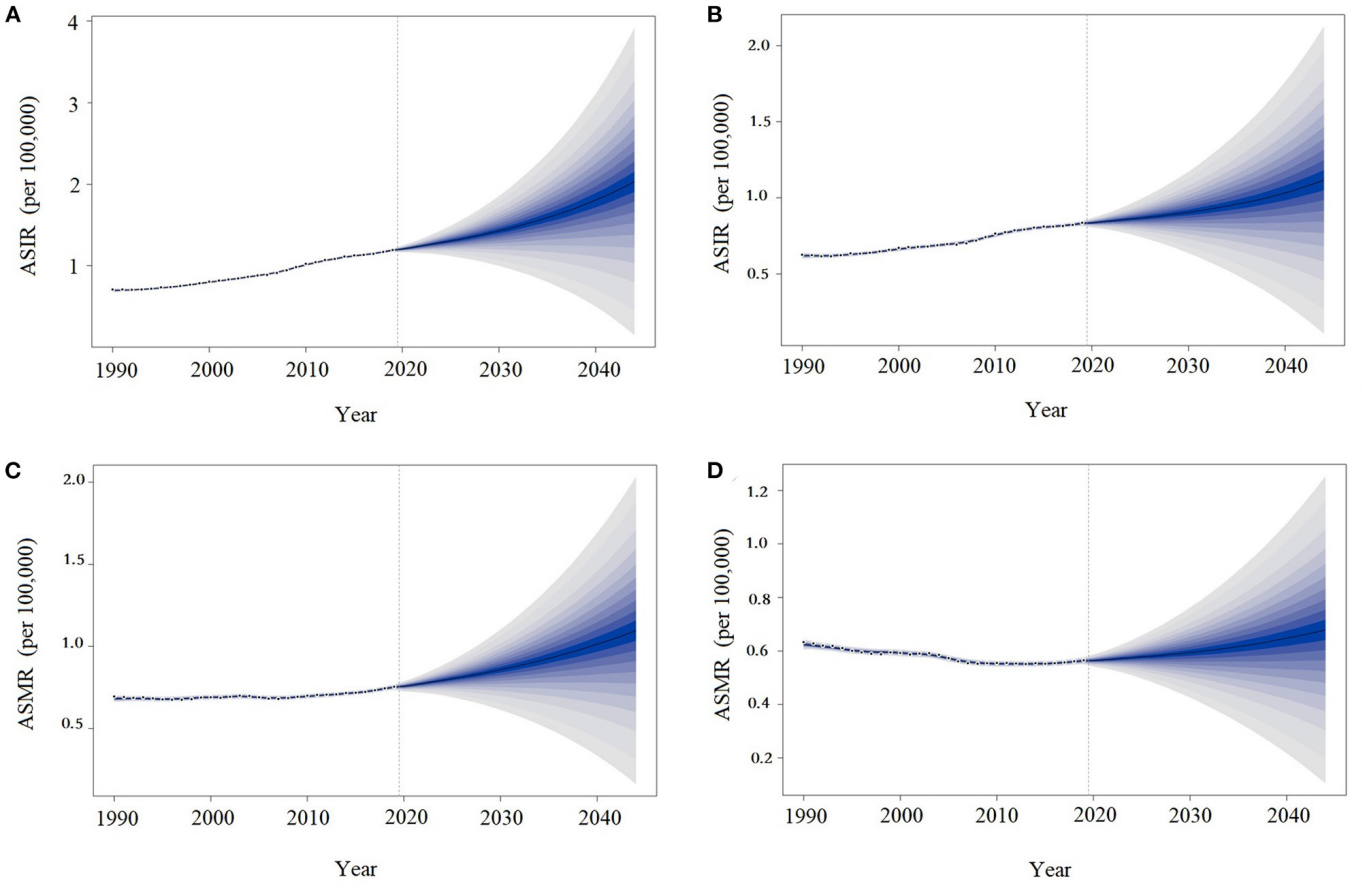
Trends in multiple myeloma incidence and death rates by sex in China: observed (dashed lines) and predicted (solid lines). The ASIR in males **(A)**, ASMR in males **(B)**, ASIR in females **(C)**, and ASMR in females **(D)**. ASIR, age-standardized incidence rates; ASMR, age-standardized death rates.

As shown in Figure 3, the ASIRs and ASMRs of MM in China will continue to increase over the next 25 years, especially among men. In 2044, the ASIRs of MM in China will reach 2.03 (95% UI: 1.15–3.91) and 1.10 (95% UI: 0.16–2.03) per 100,000 population in males and females, respectively. The ASMRs of MM in China in 2044 will be 1.12 (95% UI: 0.11–2.12) and 0.68 (95% UI: 0.11–1.25) per 100,000 population in males and females, respectively.

## Discussion

This study showed that the ASIRs and ASMRs of MM in males presented an increasing trend, whereas the ASMRs in females presented a decreasing trend from 1990 to 2019. During the observation period, the incidence rates of MM consistently exceeded the mortality rates, regardless of gender and age group. The burden of MM in males was higher than that in females. Furthermore, age and cohort effects are crucial factors of MM burden, with a high risk of MM incidence and mortality in elderly people and early-born cohort. These findings not only provide a comprehensive and up-to-date overview of temporal trends in MM burden in China over the past 30 years but also produce an important basis for strategy formulation to reduce the MM burden.

Surprising, the incidence rates increased much faster starting from 2007, especially among males, which may be largely due to improved cognition of MM and medical diagnostic techniques (17). Socioeconomic status is well-known to be significantly associated with the burden of diseases (18). The growing economy in China in recent decades has led to enhanced access to health services and more timely and accurate diagnosis of MM (19). The incidence of MM has always been higher than the mortality, may be due to advances in medical technology that have improved the detection rates and the survival rates of MM patients, and not all MM patients die from this cancer (20). For the past decades, new treatments are being developed continually, from the initial monotherapy (e.g., bortezomib, lenalidomide, and thalidomide), to the later modern combination therapy (e.g., combination of chemotherapy and autologous stem-cell transplantation), even individualized treatments for different characteristics, all of which can improve the patients' survival (21, 22). However, the mortality of males has also increased due to the influence by the increasing incidence. Notably, the mortality rates of females have been falling when the incidence of females has remained stable, which may be partly explained by the younger age of onset in female patients than in male patients (4), and age of onset is an important factor of MM burden (23). One study found that younger patients had a better prognosis than older patients for new drug use (24).

The disease burden of MM in China was much greater for males than for females. The significant difference in gender distribution of MM is consistent with previous studies (2). This finding may be due to hormonal differences between males and females, such as androgen being more likely to be a risk factor for MM than estrogen (25). Moreover, it may be partly due to the difference in exposure to risk factors between genders, such as smoking, drinking, obesity and occupational exposure, including farming, nuke industry, chemical industry and medical radiation (26). The trends of males notably showed an increasing trend in incidence and mortality rates, whereas females showed a steady trend in incidence rate and even a decreasing trend in mortality rates. Perhaps, the difference in incidence is due to different living habits, such as diet in males and females. A significant positive correlation was reported for red meat and food of animal origin men like, whereas a significant negative correlation was found for vegetables or cruciferous vegetables women like with risk of MM (27, 28). Meanwhile, the gender difference in mortality rates could be explained in part by the characteristics of the disease itself, in which the male-to-female incidence ratio was 1.5 (4), because mortality is somewhat affected by incidence. Moreover, a population-based study showed that the use of a new drug significantly reduced mortality in the group aged 70–79 years, which is mostly females, but not in the group aged 80 years and above, which is mostly males (24). This finding implied that females have a better prognosis than males.

Age is the most important risk factor for MM burden. The incidence and mortality of MM increase with age, and the risk is the highest among the elderly. Aging creates a favorable condition for the development of various chronic diseases (29). Studies have shown that aging contributes to the decline in stem cell regeneration (30). Therefore, the elderly tends to be accompanied by a decline in physiological and immune functions, and they are more likely to develop cancer (31). Moreover, the elderly have poor tolerance to side effects of antitumor therapy, and they are particularly vulnerable to toxicities during the treatment process, thus affecting the therapeutic effect and leading to poor prognosis (3). The screening of malignant tumors in the elderly should be strengthened to achieve early diagnosis and treatment to reduce MM burden.

The period effects have a significant influence on the MM incidence in males, and the risk of MM incidence increases over time. A mate-analysis showed that new diagnostic techniques in the 21st century have improved diagnostic accuracy and sensitivity of MM that was not available in earlier times (32). Another reason is that with the improvement of the medical system, cancer screening has been gradually strengthened and widely implemented, enabling more people to find the disease in time (17). Moreover, changes in the definition and diagnostic classification of the disease may also explain the rise in MM incidence. For example, from monoclonal gammopathy of undetermined significance to smoldering MM then to MM, which is a more accurate diagnosis of disease. Furthermore, China is undergoing an accelerating aging process, and studies have shown that aging increases the burden of various diseases, including MM. Although China has carried out comprehensive prevention and treatment of cancers for many years, the burden of MM is still serious due to the increase in unhealthy lifestyle, such as smoking, drinking, sedentary disease, lack of exercise and overweight (8). An aging population and the persistence of carcinogens in the environment have also notably contributed to the increased burden of MM (33). In particular, males tend to have higher levels of exposure to these risk factors than females (34).

Concerning the cohort effects, the risk of MM burden decreased with later birth cohort. In the late 19th century and early 20th century, China is experiencing various wars and natural disasters (35, 36), which increased exposure of risk factors for MM, such as ionizing radiation, chemicals, air pollution, and water pollution (8), which may explain the phenomenon that the risk of MM incidence is higher in the early-born cohort. Similarly, due to economic and medical constraints in early times, people born early cannot receive timely diagnosis and treatment, especially those with a rare disease like MM, which may account for a higher risk of death in this population (37). Fortunately, China has experienced rapid social development since the middle and late 20th century, and its socio-economic conditions, sanitation, and medical system have greatly improved (38). Studies have shown that improvements in social and occupational environments have reduced the exposure and dose of MM risk factors like ionizing radiation and medical radiation. With the spread of health knowledge, people's awareness and prevention of MM have been greatly improved (39). Moreover, the nutritional status of later-born cohort significantly improved compared with that of early-born cohort (40). Other studies have shown that health insurance status is associated with the risk of chronic disease morbidity and mortality, including MM (41). Nowadays, the increased coverage rates of three basic medical insurance in China (urban residents' medical insurance, urban workers' medical insurance, and new rural cooperative medical care) has greatly improved the medical security of later-born cohort (42). All these reasons may explain the reduced risk of MM incidence and mortality in the post-birth cohort.

The ASRs of MM incidence and mortality in China was predicted to continuous increase in the next 25 years in both sexes. On the one hand, the life expectancy in Chinese population is on the rise, indicating that the burden of MM will continue to increase with aging in the future (33). On the other hand, Chinese society is undergoing great changes, and some risk factors related to MM, such as unhealthy lifestyle, air and water pollution and occupational exposure, are changing with great uncertainty. Therefore, active and effective measures should be taken to prevent and control the risk factors of MM, such as popularizing tumor screening, reducing environmental pollution, promoting healthy diet and living habits and reducing occupational exposure, to reduce the disease burden of MM.

This study filled the gap in the long-term trend of MM disease burden in China. However, some limitations should be acknowledged. First, the GBD estimates were reconstructed on the basis of a large number of sources with different qualities, leading to potential bias from the actual data (43). Second, although the IE method used in this study has the characteristics of unbiased, efficient, asymptotic and superior estimation, the theoretical basis is complex and could not explain the practical significance of parameter estimation (44, 45). Third, because the GBD 2019 data did not include data on specific related risk factors of MM, speculations could only be conducted on the drivers of the period and cohort effects in which the incidence and mortality trends of MM were observed. Finally, age–period–cohort analysis considers a community as the observed and analyzed unit, possibly resulting in ecological fallacies. Thus, scientific assumptions concerning the causality of these temporal trends were pointed out on the basis of available data and existing literature.

## Conclusion

This study provides new evidence for the secular trend of MM incidence and mortality in China. From 1990 to 2019, the burden of MM in China is on the rise in general, and it will continue to increase in the next 25 years. A significant gender difference was observed in MM burden, which was higher in males than in females. Age and cohort effects are crucial factors of MM burden, with a high risk in elderly people and early-born cohort. A comprehensive understanding of the risk characteristics and disease pattern of MM is urgently needed, the publicity and education of relevant knowledge must be strengthened, and targeted interventions to help reduce the disease burden of MM must be urgently taken.

## Data Availability Statement

Publicly available datasets were analyzed in this study. This data can be found here: http://ghdx.healthdata.org/gbdresults-tool.

## Ethics Statement

The data in the Global Burden of Disease 2019 study database are publicly available; thus, their use was exempt from review by the Hospital Affiliated Hospital of Guangdong Medical University. The requirement for informed consent was waived.

## Author Contributions

YMZ, DDN, and JYW: study design, data collection and analysis support, and critical revision of manuscript. YMZ and DDN: data collection and analysis and drafting the manuscript. ELY, JSH, JW, and XFH: data collection. JYW: data analysis. All authors listed have made a substantial, direct, and intellectual contribution to the work and approved it for publication.

## Funding

This study was supported by Guangdong Medical University Scientific Research Fund Program (GDMUM201806).

## Conflict of Interest

The authors declare that the research was conducted in the absence of any commercial or financial relationships that could be construed as a potential conflict of interest.

## Publisher's Note

All claims expressed in this article are solely those of the authors and do not necessarily represent those of their affiliated organizations, or those of the publisher, the editors and the reviewers. Any product that may be evaluated in this article, or claim that may be made by its manufacturer, is not guaranteed or endorsed by the publisher.
