# Supplementary material for: Secular Trends in the Burden of Multiple Myeloma From 1990 to 2019 and Its Projection Until 2044 in China
Source: Front Public Health. 2022 Jul 8;10:938770. doi: 10.3389/fpubh.2022.938770 (PMC9304978; doi:10.3389/fpubh.2022.938770)
Supplement: Supplementary file 1 [file Table_1.DOCX]

**Supplementary Table 1. The sex-specific annual percentage changes (APC) of MM in the corresponding section.**

|  | Males | |  | Females | |
| --- | --- | --- | --- | --- | --- |
|  | Year | APC (%, 95% CI) |  | Year | APC (%, 95% CI) |
| Incidence | 1990–1992 | -0.40 (-1.31 – 0.51) |  | 1990–1996 | -0.35 (-0.67 – -0.02) * |
|  | 1992–2007 | 1.57 (1.53 – 1.62) * |  | 1996–2004 | 0.17 (-0.09 – 0.43) |
|  | 2007–2011 | 3.51 (3.04 – 3.98) * |  | 2004–2007 | -1.04 (-2.93 – 0.88) |
|  | 2011–2019 | 1.42 (1.32 – 1.52) * |  | 2007–2019 | 0.65 (0.53 – 0.76) * |
| Mortality | 1990–1992 | -0.66 (-1.53 – 0.22) |  | 1990–1997 | -0.90 (-1.07 – -0.72) * |
|  | 1992–2000 | 0.99 (0.87 – 1.11) * |  | 1997–2004 | -0.42 (-0.64 – -0.20) * |
|  | 2000–2007 | 0.29 (0.14 – 0.44) * |  | 2004–2007 | -2.07 (-3.35 – -0.78) * |
|  | 2007–2011 | 2.51 (2.06 – 2.96) * |  | 2007–2015 | -0.26 (-0.43 – -0.08) * |
|  | 2011–2019 | 0.65 (0.56 – 0.75) * |  | 2015–2019 | 0.54 (0.13 – 0.22) * |

* indicates the APC was significantly different from zero at the α = 0.05 level. CI, confidence interval; APC, annual percent change.
